# Genetic and metabolic mechanisms underlying webbed feet pigmentation in geese: Insights from histological, transcriptomic, and metabolomic analyses

**DOI:** 10.1016/j.psj.2025.105233

**Published:** 2025-04-29

**Authors:** Yi Liu, Kaiqi Weng, Guangquan Li, Huiying Wang, Yu Tan, Daqian He

**Affiliations:** a Shanghai Academy of Agricultural Sciences, Shanghai, China; b Hunan Wugang Tong Geese Agricultural Development Co. Ltd., Hunan, China

**Keywords:** Goose, Transcriptome, Metabolome, Webbed feet color, Mechanisms of pigmentation

## Abstract

This study systematically investigated the genetic and metabolic mechanisms underlying pigmentation in goose webbed feet by integrating histological, transcriptomic, and metabolomic analyses. Histological examinations revealed significant differences in melanin deposition among webbed feet of varying colors. Dark black webbed feet exhibited the highest melanin content, light black webbed feet showed moderate levels, and colorless webbed feet lacked detectable melanin. Transcriptomic analysis identified substantial variations in the expression levels of key genes involved in melanin biosynthesis, including *TYRP1, PMEL, DCT, TYR, OCA2, MC1R, RAB38, WNT16, CAMK2A*, and *MLANA*, between pigmented and colorless webbed feet. Notably, the *OCA2* gene exhibited significantly higher expression in dark black webbed feet compared to light black webbed feet, underscoring its pivotal role in regulating pigmentation intensity. Enrichment analysis emphasized the importance of pathways related to tyrosine metabolism, melanin production, and amino acid biosynthesis in determining pigmentation differences. Metabolomic profiling supported these findings, revealing that L-tyrosine and 5,6-dihydroxyindole-2-carboxylic acid are critical metabolites in the melanin biosynthesis pathway. Specifically, elevated levels of L-tyrosine were detected in colorless webbed feet, likely due to inhibited melanin synthesis, whereas 5,6-dihydroxyindole-2-carboxylic acid levels were highest in dark black webbed feet, reflecting active melanin production. Correlation analysis between transcriptomic and metabolomic data further validated the central role of tyrosine metabolism and melanin biosynthesis pathways in pigmentation. In conclusion, this study employed multi-omics approaches to elucidate the critical role of the *OCA2*-centered genetic-metabolic regulatory network in melanin deposition of goose webbed feet, providing important insights into the molecular mechanisms of avian pigmentation and valuable references for poultry breeding.

## Introduction

The Magang goose is a vital indigenous breed in China, renowned for its rapid growth rate and premium-quality meat. It accounts for approximately 10% of the nation’s total goose production (Li et al., 2020). This breed is distinguished by its characteristic "three black" features: a dark black head, beak, and webbed feet. While the head and beak exhibit consistent coloration, the webbed feet vary in color, ranging from light black to dark black. Among Chinese consumers, dark webbed feet are considered key indicators of purebred Magang geese, making them a highly desirable trait that significantly influences consumer preferences and market value. Despite their importance, the genetic and biochemical mechanisms underlying webbed feet color variation remain largely unexplored. The Hungarian white goose, a representative species of European geese, is characterized by its pure white plumage and non-pigmented webbed feet, which contrasts with the pigmented webbed feet of the Magang goose. This pronounced phenotypic difference serves as a valuable basis for narrowing down the investigation of candidate genes associated with pigment synthesis. Consequently, this study aimed to investigate the genetic and metabolic mechanisms underlying webbed feet skin pigmentation in geese by comparing various pigmented types of webbed feet.

In poultry, external traits such as feather color, shank color, beak color, and webbed feet color are categorized as packaging traits (Hamadani et al., 2014; Zhang et al., 2023). Among these traits, feather color has garnered significant attention. Extensive research has shown that variations in feather color across poultry species are primarily attributed to melanin and carotenoids, with melanin playing the dominant role (Galván and Solano, 2016). The genetic basis of melanin pigmentation has been widely studied, identifying several candidate genes involved in melanin synthesis, distribution, and deposition. As a rate-limiting enzyme for melanin synthesis, tyrosinase (*TYR*) mutations have been shown to cause feather color variation in poultry (Xu et al., 2013; Wang et al., 2024). Additionally, receptor protein tyrosinase-related protein 1 (*TYRP1*), which is associated with tyrosinase-related processes, plays a critical role in determining poultry feather color. Mutations in the *TYRP1* gene can lead to significant alterations in feather pigmentation (Li et al., 2018; Kinoshita et al., 2024). As a key regulator of melanin synthesis and tyrosinase activity, microphthalmia associated transcription factor (*MITF*) plays a key regulatory role in the formation of feather color in poultry (Wang et al., 2017; Zhou et al., 2018). Furthermore, genes such as melanocortin-1 receptor (*MC1R*) (Nam et al., 2021; Schwochow et al., 2021; Liu et al., 2024), endothelin receptor B2 (*EDNRB2*) (Kinoshita et al., 2014; Li et al., 2015), dopachrome tautomerase (*DCT*) (Sultana et al., 2017), and agouti signaling protein (*ASIP*) (Yang et al., 2019; Lin et al., 2024) have also been identified as key regulators influencing poultry feather coloration. These genes play crucial roles in the melanogenesis pathway, significantly influencing feather color and other pigmentation traits.

In contrast, the coloration of bare parts, such as the beak, legs, and webbed feet, exhibits a more limited range of variability compared to feathers. Similar to feathers, the pigmentation of bare parts in birds is influenced by pigments and structural changes or alterations in blood flow beneath the skin, which can reveal or conceal underlying colors (Iverson et al., 2017). In unfeathered regions, the primary pigments are carotenoids and melanin, with carotenoid-based coloration being more prominent compared to feathers (Price-Waldman and Stoddard, 2021). For instance, the recessive allele (W*Y) results in the deposition of yellow carotenoids in domestic chicken skin, imparting a yellow hue (Eriksson et al., 2008). Mutations in β-carotene oxygenase 2 (*BCO2*) are responsible for variations in carotenoid concentration in chicken skin (Wang et al., 2023) and bird beaks (Gazda et al., 2020; Enbody et al., 2021), leading to color polymorphism. Recent studies have identified beta-carotene dioxygenase 2 (*BCDO2*), located on chromosome GGA24, as the principal regulatory gene for shank skin color traits in chickens (Jin et al., 2016). In ducks, candidate genes such as *MITF, EDNRB2*, and members of the Solute Carriers (*SLC*) superfamily have been implicated in regulating spotted tibia coloration (Guo et al., 2022). These findings highlight significant differences in the genetic mechanisms governing pigmentation in feathers versus unfeathered regions such as beaks, shanks, and webbed feet. However, the genetic mechanisms underlying webbed feet coloration in geese remain largely unexplored.

This study investigates the correlation between webbed feet skin color variation and the presence of melanin in geese. By examining the distribution patterns of melanocytes across varying webbed feet pigmentation and analyzing transcriptomic and metabolomic data, this study aims to elucidate the genetic mechanisms underlying webbed feet coloration. The findings not only provide a robust theoretical foundation for selecting and improving phenotypic traits in geese but also contribute to a deeper understanding of pigmentation mechanisms in poultry. Moreover, these insights have the potential to enhance the economic value of high-quality meat geese, such as the Magang breed, offering novel perspectives for breeding selection strategies.

### Materials and methods

#### Animal ethics

The research fully complied with the 2017 *Regulations on the Management of Laboratory Animals* issued by the State Council of the People's Republic of China. Ethical approval was granted by the Animal Ethics Committee of the Shanghai Academy of Agricultural Sciences (Shanghai, China) under approval code SAASPZ0522046. All experimental procedures were conducted in strict accordance with ethical guidelines for animal research.

#### Animals and Sample collection

**Fig. 1:**
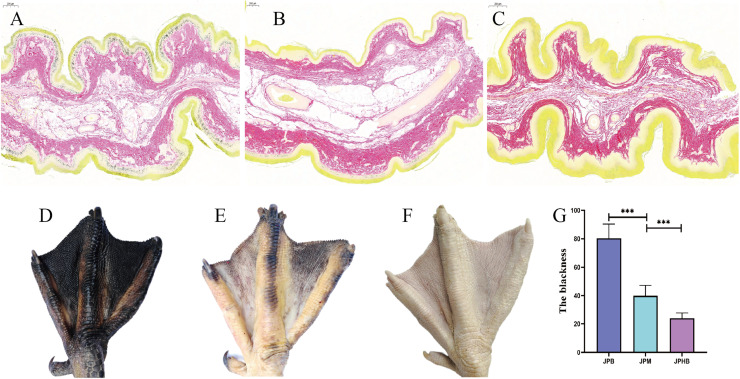
Illustration of flipper color, sampling locations and melanin distribution in flippers with varying pigmentation. (A) Representative histological images of dark black flippers. (B) Representative histological images of light black flippers. (C) Representative histological images of colorless flippers. (D) Representative images of dark black flippers. (E) Representative images of light black flippers. (F) Representative images of colorless flippers. (G) The blackness of flippers with varying pigmentation. Melanin is indicated using red line segments and font markings. The key structures of the skin are highlighted using green line segments and font markings. The red box indicates the location of sample collection. (JPB: dark black flippers; JPM: light black flippers; JPHB: colorless flippers; n=7).

The experiment was conducted at Hunan Wugang Tong Geese Agricultural Development Co., Ltd., located in Wugang City, Hunan Province, China. For this study, 70-day-old Magang geese and Hungarian White geese, all hatched simultaneously and raised under identical conditions, were selected. The sample included seven Magang geese with dark black webbed feet (JPB) (n=7), seven with light black webbed feet (JPM) (n=7), and seven Hungarian White geese with colorless webbed feet (JPHB) (n=7) (Fig. 1).

The geese were humanely euthanized using CO₂ inhalation, followed by cervical dislocation performed by a certified professional. Immediately after euthanasia, webbed feet samples (1 cm × 1 cm) were collected from the middle and outer toes of each goose. The samples were divided into four portions: one was fixed in 4% paraformaldehyde at room temperature for histological analysis, while the other two were rapidly frozen in liquid nitrogen and stored in 2 ml cryovials at -80°C for transcriptomic and metabolomic analyses. The last sample was fixed with in situ hybridization fixative (4% paraformaldehyde without RNA enzymes). The webbed feet color and sampling locations are illustrated in Fig. 1D-F. The degree of blackness in the webbed feet was quantitatively assessed using the DS700C colorimeter (CHNSpec, Zhejiang, China).

#### Histological Analysis

After fixation for over 24 hours, the webbed feet samples were carefully retrieved from the fixative solution. Using a scalpel, the target tissue was meticulously trimmed. The trimmed tissues underwent dehydration, wax infiltration, embedding, and sectioning into 4 µm thick slices for silver nitrate staining.

The paraffin sections were deparaffinized by sequential immersion in Xylene I and Xylene II for 20 minutes each. Hydration was performed through two consecutive treatments with anhydrous ethanol (5 minutes each), followed by immersion in 75% ethanol for 5 minutes. After hydration, the sections were thoroughly rinsed 3–5 times with tap water and distilled water.

Each slide was stained using the Masson Fontana Stain Kit (Wuhan Servicebio Technology Co., Ltd., Wuhan, China) and mounted with neutral resin. The stained webbed feet sections were examined under a Nikon Eclipse E100 microscope (Nikon, Tokyo, Japan), and images were captured using the NIKON DS-U3 imaging system (Nikon, Tokyo, Japan).

### Total RNA isolation and Transcriptome sequencing

RNA extraction from webbed feet samples was conducted using Trizol reagents (Invitrogen, Carlsbad, CA, USA) following the manufacturer's protocol. RNA concentration and purity were assessed using a NanoDrop spectrophotometer (Thermo Fisher Scientific Inc., Waltham, MA, USA), and RNA integrity was confirmed via RNA-specific agarose-gel electrophoresis.

High-quality RNA (OD260/280 > 2.0, OD260/230 > 2.0) was used to construct cDNA libraries, with 3 µg of RNA processed using the NEBNext Ultra II RNA Library Prep Kit (New England Biolabs Inc., Ipswich, MA, USA). The library preparation involved RNA fragmentation, end repair, and adapter ligation, optimized for Illumina sequencing platforms. Library fragments were purified using the AMPure XP system (Beckman Coulter, Brea, CA, USA), targeting a fragment size range of 400–500 bp.

Adapter-ligated fragments were amplified through 15 cycles of PCR using Illumina-supplied primers. The amplified products were further purified using the AMPure XP system and quantified using an Agilent 2100 bioanalyzer (Agilent Technologies, Santa Clara, CA, USA). The final libraries were sequenced on the NovaSeq 6000 platform (Illumina, USA), with sequencing services provided by Shanghai Personal Biotechnology Co. Ltd., China.

#### Transcriptome Analysis

To ensure high-quality sequencing results, raw data underwent stringent quality control and filtering using fastp (version 0.22.0). This process removed sequences contaminated by 3′ adapter artifacts and reads with an average quality score below Q20. Preprocessed reads were aligned to the reference genome (GenBank accession: GCF_002166845.1, available at NCBI) using HISAT2 (version 2.1.0) (Kim et al., 2019).

Gene expression levels were quantified by counting reads mapped to each gene using HTSeq (version 0.9.1) (Anders et al., 2014), and normalized using the FPKM method. Differentially expressed genes (DEGs) were identified with DESeq2 (version 1.38.3) (Wang et al., 2009) based on the criteria |log2FoldChange| > 1 and adjusted P-value < 0.05. Bidirectional clustering of DEGs was performed using the R package Pheatmap (version 1.0.12) (Love et al., 2014), grouping genes into distinct clusters with similar expression patterns. Enrichment plots were generated to visualize significantly enriched terms within each cluster.

Gene Ontology (GO) enrichment analysis was conducted with topGO (version 2.50.0) (Ashburner et al., 2000) using a hypergeometric test, defining significant enrichment as adjusted P-value < 0.05. Kyoto Encyclopedia of Genes and Genomes (KEGG) pathway enrichment analysis was performed using clusterProfiler (version 4.6.0) (Yu et al., 2012) to identify pathways with adjusted P-value < 0.05. Additionally, gene set enrichment analysis (GSEA) was conducted using GSEA software (version 4.1.0), and the resulting GSEA enrichment pathway map was generated.

### Metabolite extraction and detection

50 mg of tissue was homogenized in 2 mL centrifuge tubes with steel beads and 200 μL ice-cold water at 55 Hz for two 60-second cycles. Methanol-acetonitrile (1:1, v/v, 800 μL) was added, and the samples were sonicated for 30 minutes, frozen at −20°C for 30 minutes, and centrifuged at 12,000 rpm for 10 minutes at 4°C. The supernatant (800 μL) was collected, vacuum-dried, reconstituted in 150 μL of 50% methanol with 5 ppm 2-chlorophenylalanine, vortexed for 30 seconds, centrifuged, and filtered through a 0.22-μm filter. UHPLC analysis was performed on a Vanquish Flex system with an HSS T3 column (2.1 × 100 mm, 1.8 μm) at 40°C and 0.4 mL/min flow rate. The mobile phase consisted of 0.1% formic acid in water (A) and acetonitrile (B). The gradient program was: 0–1 min (5% B), 7–8 min (95% B), and 8.1–13 min (5% B). Mass spectrometry was conducted using an Orbitrap Exploris 120 with HESI in positive and negative modes (±3.5/−3.0 kV). Instrument parameters included sheath gas flow at 40, auxiliary gas flow at 15, and capillary temperature at 325°C. Full MS scans were acquired at 60,000 resolution over 100–1000 m/z. Data-dependent MS² scans were performed on the top four ions with 30% HCD collision energy and 8-second dynamic exclusion. Pooled QC samples (10–20 μL per sample) were injected before and after every 5–10 samples to ensure stability.

### Metabolome analysis

To ensure high-quality data acquisition, raw data generated by the mass spectrometer were systematically evaluated and filtered using Compound Discoverer™ 3.3 (version 3.3.2.31, Thermo Fisher Scientific, Waltham, MA, USA). This process included background noise reduction and the elimination of low-quality peaks, followed by normalization of the total peak area. Metabolite identification was performed by referencing an in-house database and several authoritative online databases, including mzCloud (https://www.mzcloud.org/), LIPID MAPS (https://www.lipidmaps.org/), HMDB (https://hmdb.ca/), MoNA (https://mona.fiehnlab.ucdavis.edu/), and the NIST_2020_MSMS spectral library. Identification criteria included an MS1 mass tolerance of 15 ppm and an MS2 match factor threshold of 50.

Visualization of metabolite abundances was achieved through expression abundance density plots and violin plots generated using ggplot2 (version 3.4.1). Differentially expressed metabolites (DEMs) were subjected to comprehensive bioinformatics analyses. Clustering analysis of abundance values was conducted using the Pheatmap package (version 1.0.12) in R, resulting in heatmap visualizations. Venn diagrams and UpSet plots were created with the VennDiagram (version 1.7.3) and UpSetR (version 1.4.0) packages, respectively, to illustrate overlaps between groups. Correlation analysis was performed using the corrplot package (version 4.0.3). Boxplots and violin plots, generated with ggplot2, depicted metabolite abundance changes across experimental groups. Functional roles of DEMs were explored through KEGG pathway enrichment analysis using the clusterProfiler package (version 4.6.0), identifying significantly enriched metabolic pathways.

### Analysis of the correlations between the transcriptome and metabolome

For the integrated analysis of metabolomics and transcriptomics, results from both datasets were consolidated, and correlation and O2PLS analyses were conducted. Differential metabolites and transcripts were identified using Metscape 2, and enzyme-related transcripts corresponding to these metabolites were extracted from the KEGG database (https://www.kegg.jp/dbget-bin/www_bfind?compound). Pearson correlation coefficients between DEMs and transcripts were calculated using R's cor function, and a correlation network was constructed.

Additionally, DEMs and all transcripts were mapped to the KEGG pathway database to identify shared pathways, focusing on genes with p-values < 0.05 and metabolites with VIP values > 1 and p-values < 0.05. Metabolic pathways involving these DEMs and their corresponding transcripts were visualized using the pathview package in R. A correlation network for shared pathways was generated, including only correlations where the coefficient between genes and metabolites exceeded 0.8.

### Validation of DEGs results using qRT-PCR

To validate the DEGs across various webbed feet colors, eight candidate genes significantly associated with melanin were selected for quantitative real-time PCR (qRT-PCR) analysis. Primers for these genes were designed based on previously assembled sequences using Oligo 6.0 software. Detailed information on these genes and their corresponding primers is provided in additional table 1. The qRT-PCR experiments were performed using the SYBR Premix Ex Taq™ II kit (RR820A, Takara, Dalian, China) on an Applied Biosystems 7500 Fast Real-Time PCR System (7500, ABI, USA). Each reaction mixture contained 1 µL of cDNA template, 10 µL of SYBR Premix Ex Taq, 0.4 µL of Rox Reference Dye (II), 7.4 µL of nuclease-free water, and 0.6 µL of each gene-specific primer. The amplification protocol consisted of an initial denaturation at 95°C for 2 minutes, followed by 40 cycles of denaturation at 95°C for 30 seconds, annealing at 58°C for 30 seconds, and extension at 72°C for 30 seconds. A final melting curve analysis was conducted to confirm the specificity of the amplified products. Relative expression levels of the candidate genes were calculated using the threshold cycle (Ct) values, normalized to *GAPDH*, and analyzed using the 2^-ΔΔCt^ method.

### Fluorescence In Situ Hybridization (FISH)

Webbed feet tissues were fixed in situ hybridization fixative for a minimum of 12 hours at 4°C, followed by sequential dehydration using a graded ethanol series (75% to 85% to 100%), xylene clearing, and paraffin embedding. Sections, cut to a thickness of 4 μm, were baked at 62°C for 2 hours, dewaxed using BioDewax solution, rehydrated through a reverse graded ethanol series (100% to 85% to 75%), and equilibrated in DEPC-treated water. The tissue sections were placed in a citric acid buffer (pH 6.0) repair solution and incubated in a water bath at 90 degrees Celsius for 48 minutes. Subsequently, sections were digested with 20 µg/ml proteinase K at 40 °C for 10 minutes, followed by three washes with PBS for 5 minutes each. The sections were incubated with pre-hybridization solution at 37 °C for 1 hour, then hybridized with target-specific probes (Additional table 2) in a humidified chamber at 42°C overnight. Post-hybridization washes consisted of sequential SSC buffer treatments (2×, 1×, and 0.5×) optimized with formamide to minimize nonspecific binding. Signal amplification was achieved using branched DNA probes incubated at 40°C for 45 minutes, followed by the application of tyramide-conjugated fluorophores at a dilution of 1:200 and an incubation temperature of 42°C for 3 hours. Nuclei were counterstained with DAPI at room temperature for 8 minutes, mounted in an antifade medium, and imaged using the Nikon Eclipse ci fluorescence microscope (Nikon, Tokyo, Japan).

### Statistical analysis

Data management and analysis of candidate gene expression levels were performed using Excel 2007. Statistical analysis was conducted with SPSS 26.0 software, applying one-way ANOVA, followed by post-hoc comparisons using Duncan's test. Significance levels were set at P < 0.05 for significant differences, P < 0.01 for highly significant differences, and P > 0.05 for non-significant differences.

## Results

### Distribution of melanin in webbed feet with varying pigmentation

Histological analysis using silver nitrate staining revealed significant differences in melanin distribution and quantity across geese with different webbed feet colors. Dark black webbed feet exhibited extensive melanin deposition in the epidermal layer, with densely packed melanin granules forming distinct pigmented regions (Fig. 1A). In contrast, light black webbed feet showed significantly reduced melanin, with only a few scattered granules (Fig. 1B). No melanin was detected in the epidermal layer of colorless webbed feet, indicating a complete absence of melanin synthesis or deposition (Fig. 1C). In addition, the blackness of dark black webbed feet is considerably higher compared to that of light black webbed feet, whereas the blackness of colorless webbed feet remains at the lowest level (Fig. 1G). These results suggest that melanin presence and quantity are crucial in determining the color of goose webbed feet.

### Analysis of DEGs in webbed feet with varying pigmentation

**Fig. 2:**
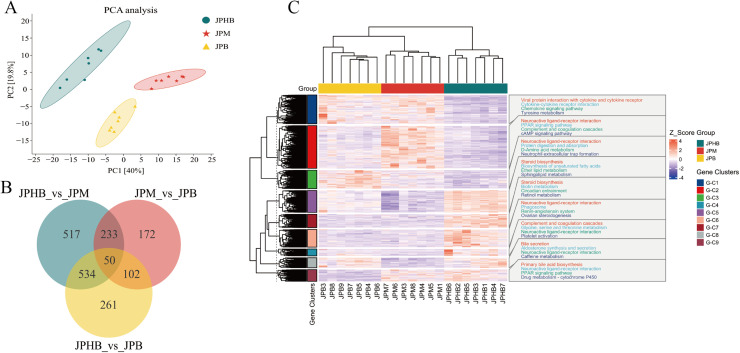
Transcriptome analysis of goose webbed feet tissues with varying pigmentation. (A) Principal component analysis (PCA) of mRNA-Seq samples. (B) Venn diagram illustrating the overlap of differentially expressed genes (DEGs) between groups. (C) Hierarchical clustering analysis of DEGs and enriched pathways. JPB: dark black webbed feet; JPM: light black webbed feet; JPHB: colorless webbed feet; n=7.

To investigate the transcriptomic characteristics of goose webbed foot tissues with varying colors, this study conducted an mRNA-Seq analysis of goose webbed foot tissues. The raw data have been deposited in the Sequence Read Archive (SRA) database (accession number: PRJNA1224235). Principal Component Analysis (PCA) revealed distinct transcriptomic profiles for each webbed feet type, indicating significant gene expression differences (Fig. 2A). DEGs were identified through pairwise comparisons, using a threshold of |log2 fold change| > 1 and P-adjust < 0.05. The Venn diagram showed 584 commonly differentially expressed genes between dark black and colorless webbed feet, and light black and colorless webbed feet. Additionally, gene expression analysis revealed distinct patterns across pigmentation intensities: 283 differentially expressed genes (DEGs) were shared between dark black and light black webbed feet, 152 DEGs overlapped between light black and colorless feet, with a core set of 50 DEGs consistently differentially expressed across all three pigmentation comparisons (Fig. 2B). The clustering heatmap revealed consistent clustering patterns among biological replicates of each webbed feet type (Fig. 2C). Enrichment analysis identified pathways related to webbed feet pigmentation, such as tyrosine metabolism, in the G-C1 cluster (Fig. 2C).

### Analysis of GO and KEGG enrichment in DEGs

**Fig. 3:**
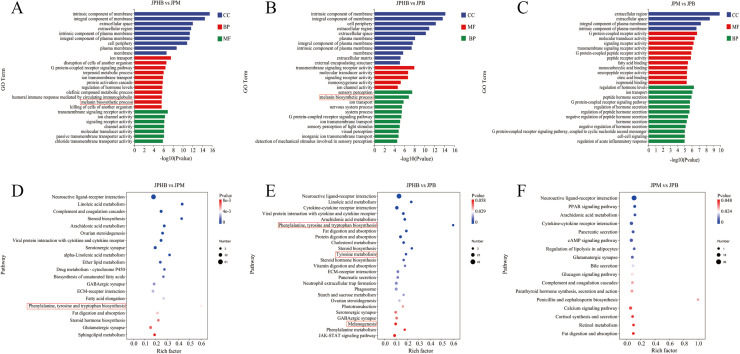
GO and KEGG enrichment analysis of differentially expressed genes (DEGs) in goose webbed feet tissues with varying pigmentation. (A) GO classification map comparing JPHB and JPM. (B) GO classification map comparing JPHB and JPB. (C) GO classification map comparing JPM and JPB. (D) Enriched KEGG pathways comparing JPHB and JPM. (E) Enriched KEGG pathways comparing JPHB and JPB. (F) Enriched KEGG pathways comparing JPM and JPB. JPB: dark black webbed feet; JPM: light black webbed feet; JPHB: colorless webbed feet; n=7.

To investigate the biological significance of DEGs and their relationship with webbed feet pigmentation, GO and KEGG enrichment analyses were conducted. GO term enrichment revealed significant associations with molecular function (MF), cellular component (CC), and biological process (BP) categories (Figs. 3A-C). Specifically, DEGs between colorless and light black webbed feet were enriched in terms related to the membrane's intrinsic and integral components, as well as extracellular space (Fig. 3A). DEGs between colorless and dark black webbed feet were associated with the intrinsic and integral components of the membrane and the cell periphery (Fig. 3B). In contrast, DEGs between light black and dark black webbed feet were primarily related to the extracellular region, extracellular space, and plasma membrane components (Fig. 3C). Notably, the melanin biosynthesis pathway, highlighted in red, was significantly enriched in comparisons between colorless and pigmented webbed feet (light black and dark black) (Fig. 3 A and B), but not between light black and dark black webbed feet (Fig. 3C). The enrichment results suggest that the melanin biosynthesis pathway might be closely associated with webbing pigmentation, though further experimental validation is necessary to confirm causality. KEGG analysis revealed that neuroactive ligand-receptor interaction, linoleic acid metabolism, and complement and coagulation cascades were the most enriched pathways between colorless and light black webbed feet (Fig. 3D), while neuroactive ligand-receptor interaction, linoleic acid metabolism, and cytokine-cytokine receptor interaction were enriched between colorless and dark black webbed feet (Fig. 3E). For the light black vs. dark black comparison, the most enriched pathways included neuroactive ligand-receptor interaction, peroxisome proliferator-activated receptor (PPAR) signaling, and arachidonic acid metabolism (Fig. 3F). Similar to GO analysis, key melanin-related pathways, including phenylalanine, tyrosine, and tryptophan biosynthesis, tyrosine metabolism, and melanogenesis, were significantly enriched in comparisons between colorless and pigmented webbed feet (Fig. 3, Fig. 3), but not between light black and dark black webbed feet (Fig. 3F). These findings further support the association between goose webbed feet pigmentation and specific metabolic pathways.

### Identification of Key Regulatory Genes in melanin biosynthesis process

**Fig. 4:**
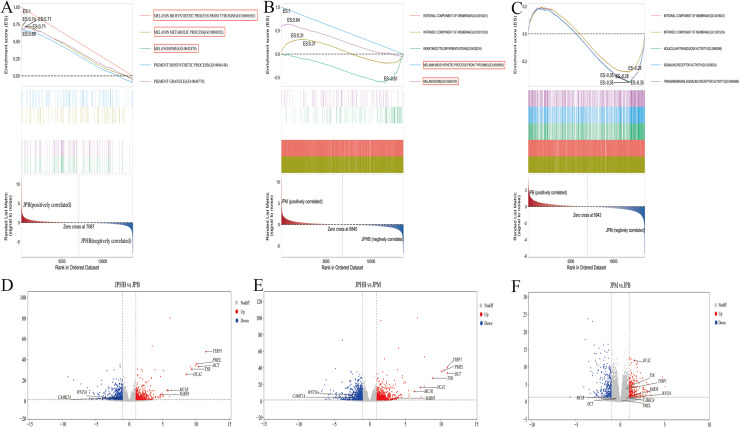
GSEA multi-pathway enrichment analysis and volcano plots of DEGs in goose webbed feet tissues with varying pigmentation. (A) GSEA multi-pathway enrichment comparing JPHB and JPM. (B) GSEA multi-pathway enrichment comparing JPHB and JPB. (C) GSEA multi-pathway enrichment comparing JPM and JPB. (D) Volcano plot of DEGs comparing JPHB and JPM. (E) Volcano plot of DEGs comparing JPHB and JPB. (F) Volcano plot of DEGs comparing JPM and JPB. JPB: dark black webbed feet; JPM: light black webbed feet; JPHB: colorless webbed feet; n=7.

**Fig. 5:**
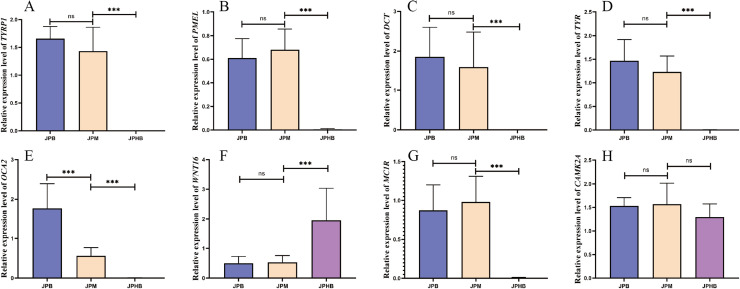
The expression of genes in various pigmented webbed feet. A-H represent the expression trends of genes (*TYRP1, PMEL, DCT, TYR, OCA2, WNT16, MC1R* and *CAMK2A*) in JPB, JPM and JPHB. (JPB: dark black webbed feet; JPM: light black webbed feet; JPHB: colorless webbed feet; n=7; ns*p* > 0.05, ***p<0.001).

GSEA multi-pathway enrichment analysis revealed the overall expression trends of functional gene sets within the pathways. Figs. 4A-C show the top five significantly enriched pathways. In the comparison between colorless and light black webbed feet, the top three pathways were all melanin synthesis-related: melanin biosynthetic process from tyrosine, melanin metabolic process, and melanosome formation, highlighted in red (Fig. 4A). In the comparison between colorless and dark black webbed feet, melanin-related pathways, such as melanin biosynthetic process from tyrosine and melanosome formation, ranked fourth and fifth, respectively (Fig. 4B). These pathways showed higher expression levels in pigmented webbed feet (both dark and light black) compared to colorless webbed feet. However, no melanin-related pathways were significantly enriched in the comparison between light black and dark black webbed feet (Fig. 4C). Nine key genes involved in these pathways were selected for differential expression analysis. The volcano plot showed that genes such as *TYRP1*, premelanosome protein gene (*PMEL*), *DCT, TYR*, oculocutaneous albinism type II (*OCA2*), *MC1R*, and ras-related protein Rab-38 (*RAB38*) were significantly upregulated in pigmented webbed feet compared to colorless webbed feet, while Wnt family member 16 (*WNT16*) and calcium/calmodulin-dependent protein kinase II alpha (*CAMK2A*) were downregulated (Fig. 4D). The expression profiles of these genes in various pigmented webbed feet except *CAMK2A* were found to be highly consistent with the transcriptome analysis data obtained via qRT-PCR (Fig. 5), confirming the reliability of our findings. In contrast, no significant gene expression differences were observed between light black and dark black webbed feet, except for *OCA2*, which was significantly upregulated in dark black webbed feet. These results were consistent in the comparison between colorless and light black webbed feet via qRT-PCR (Fig. 5). Furthermore, we employed in situ hybridization to investigate the expression pattern and localization of the *OCA2* gene in webbed feet of varying pigmentation (Fig. 8). The results showed that the expression pattern of *OCA2* gene in webbed feet of varying pigmentation was consistent with the transcriptome and qRT-PCR results, and the expression was located in the skin of the webbed feet. These findings suggest that goose webbed feet pigmentation is closely linked to melanin levels, with *OCA2* expression correlating with pigmentation intensity.

### Metabolome Analysis and Identification of Key metabolite in melanin biosynthesis process

**Fig. 6:**
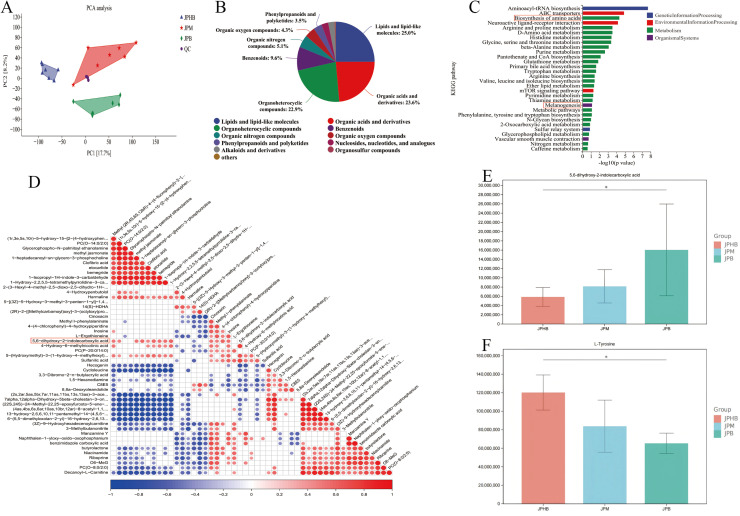
Metabolome analysis of goose webbed feet tissues with varying pigmentation. (A) Principal component analysis (PCA) of metabolomic profiles, showing the separation of samples based on pigmentation groups. (B) Classification and identification of metabolites. (C) KEGG enrichment analysis of differentially abundant metabolites. (D) Correlation analysis of differentially abundant metabolites, illustrating the relationships between key metabolites. (E-F) Relative abundances of L-tyrosine and 5,6-dihydroxy-2-indolecarboxylic acid across the three webbed feet types. JPB: dark black webbed feet; JPM: light black webbed feet; JPHB: colorless webbed feet; n=7.

To investigate metabolic differences among goose webbed feet of varying pigmentation, metabolomic sequencing was performed on dark black, light black, and colorless goose webbed feet. Metabolome analysis was performed on 21 samples, with raw data deposited in the national geophysical data center (NGDC) (accession number: OMIX009040). PCA revealed distinct clusters for each tissue type, indicating significant metabolic heterogeneity (Fig. 6A). The small distance between QC samples suggests high-quality data. Metabolite classification showed that Lipids and lipid-like molecules (25.0%), Organic acids and derivatives (23.6%), and Organoheterocyclic compounds (22.9%) were the most abundant classes (Fig. 6B). KEGG pathway enrichment analysis indicated that differentially abundant metabolites were primarily enriched in pathways such as Aminoacyl-tRNA biosynthesis, ABC transporters, and Biosynthesis of amino acids (Fig. 6C). Notably, two metabolites, MP4939 (L-tyrosine) and MP4700 (5,6-dihydroxy-2-indolecarboxylic acid), were significantly enriched in the biosynthesis of amino acids and melanogenesis pathways. Correlation analysis showed significant correlations for 5,6-dihydroxy-2-indolecarboxylic acid with several metabolites, while no such correlations were observed for L-tyrosine (Fig. 6D). Fig. 5, Fig. 5 show the relative abundances of L-tyrosine and 5,6-dihydroxy-2-indolecarboxylic acid across the three webbed feet types. 5,6-dihydroxy-2-indolecarboxylic acid was most abundant in dark black webbed feet and least in colorless webbed feet (p<0.05) (Fig. 6E), while L-tyrosine was least abundant in dark black webbed feet and most abundant in colorless webbed feet (p<0.05) (Fig. 6F).

### Analysis of the correlations between Transcriptome and Metabolome

**Fig. 7:**
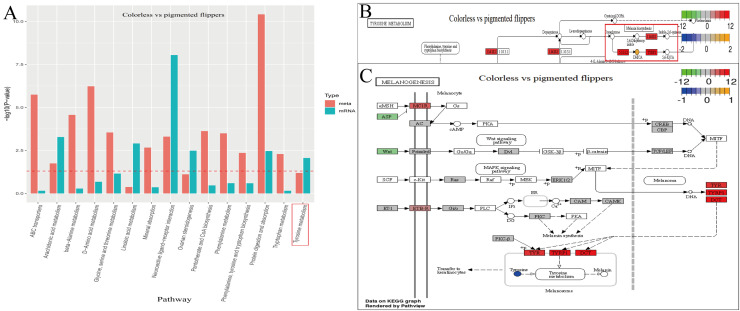
Transcriptome-metabolome correlation analysis. (A) KEGG pathway analysis highlighting differentially abundant metabolites and DEGs between colorless (JPHB) and pigmented (JPB/JPM) webbed feet. (B) Tyrosine metabolism pathway, showing key metabolites (5,6-dihydroxy-2-indolecarboxylic acid) and associated DEGs involved in pigmentation regulation. (C) Melanogenesis pathway, illustrating critical metabolites (L-tyrosine) and DEGs linked to melanin synthesis. JPB: dark black webbed feet; JPM: light black webbed feet; JPHB: colorless webbed feet; n=7.

The differentially abundant metabolites (VIP > 1, p < 0.05) and DEGs (p < 0.05) between colorless and pigmented webbed feet, as well as between light black and dark black webbed feet, to the KEGG pathway database, were mapped. A correlation analysis of genes and metabolites within shared pathways resulted in a correlation network diagram. In the comparison between colorless and pigmented webbed feet, genes and metabolites were linked to melanin synthesis pathways, such as tyrosine metabolism (Fig. 7A, red box). In contrast, no significant genes or metabolites related to melanin synthesis were identified in the comparison between light and dark black webbed feet. In the tyrosine metabolism pathway, 5,6-dihydroxyindole-2-carboxylic acid and *TYRP1* gene expression were significantly elevated in pigmented webbed feet (Fig. 7B), indicating a link to melanin synthesis regulation. In the melanogenesis pathway, tyrosine levels were significantly higher in pigmented webbed feet, while the expression of *TYR, TYRP1*, and *DCT* genes showed the opposite trend (Fig. 7C). No melanin-related pathways were found in the comparison between light and dark black webbed feet.

**Fig. 8:**
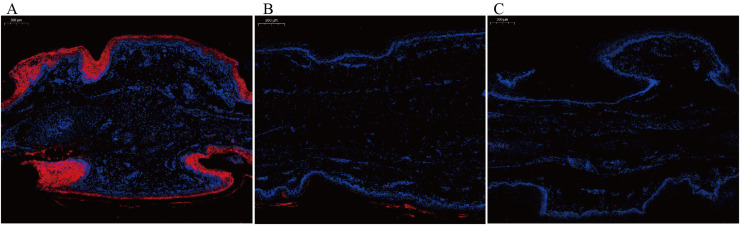
FISH for *OCA2* and relative *OCA2* expression in webbed feet with varying pigmentation. Representative images of FISH for the *OCA2* gene were exhibited in three distinct color webbed feet, with a scale bar of 200μm. The localization of *OCA2* is marked in red. (A) Representative images of dark black webbed feet (JPB). (B) Representative images of light black webbed feet (JPM). (C) Representative images of colorless webbed feet (JPHB).

## Discussion

This study aimed to explore the distribution of melanin in goose webbed feet and its underlying molecular and metabolic mechanisms. Through histological, transcriptomic, and metabolomic analyses, significant differences in melanin deposition and gene expression among dark black, light black, and colorless webbed feet were identified. The results suggest that goose webbed feet pigmentation is primarily influenced by the melanin biosynthesis pathway and related metabolic processes. This research provides valuable insights into the genetic and metabolic mechanisms of pigmentation in non-feathered regions of poultry, an area that remains underexplored.

Histological analysis revealed that melanin deposition was highest in dark black webbed feet, followed by light black webbed feet, while colorless webbed feet lacked melanin. This absence suggests a complete inhibition of melanin biosynthesis in colorless webbed feet, supporting previous research on the role of melanin in avian appendage pigmentation (Wang et al., 2021). These findings highlight the critical role of melanin deposition in determining webbed feet color variation in geese.

Melanin, a primary pigment determining phenotypic traits like skin and webbed feet coloration, is regulated by a complex genetic network (Price-Waldman and Stoddard, 2021; Roulin and Ducrest, 2013). The study identifies *TYRP1, DCT, TYR, PMEL, MLANA*, and *RAB38* as upregulated in pigmented webbed feet, consistent with their canonical roles in melanosome biogenesis, melanin synthesis, and trafficking. For instance, mutations in *TYR*, the rate-limiting enzyme for melanin synthesis, and its associated receptor protein *TYRP1*, which regulates tyrosinase-related processes, have been shown to critically influence feather color variation in poultry by altering pigmentation patterns (Wang et al., 2024; Kinoshita et al., 2024). The upregulation of *RAB38* and *MLANA* aligns with their roles in melanosome maturation and transport, as Rab GTPases regulate vesicle trafficking (Fukuda et al., 2021), while MLANA is a transmembrane protein specifically expressed in melanocytes (Vincek and Rudnick, 2022). Previous studies on duck beak (Pan et al., 2023) and skin color (Wang et al., 2021) have demonstrated that the *TYRP1, DCT, TYR, OCA2, RAB38, MLANA*, and *EDNRB* genes are significantly associated with the melanin synthesis pathway. The expression levels of these genes were notably higher in dark beaks compared to light beaks; a finding consistent with the gene expression patterns observed in webbed feet. However, while previous research identified the *MITF* and *EDNRB* genes as key determinants of beak color (Guo et al., 2022) and feather color (Zhou et al., 2022; Guo et al., 2022), this study found no significant differences in the expression of *MITF* and *EDNRB* genes across the three different webbed feet colors. However, the results of this study suggest that *OCA2* may play a crucial role in regulating webbed feet pigmentation. This suggests that while beak, feather, and webbed feet color are all related to melanin synthesis, there may be differences in their regulatory mechanisms. In the process of melanin synthesis, the pH value of the organelle known as the melanosome plays a critical role. This is because tyrosinase, a key enzyme in melanin synthesis, has its activity directly influenced by the pH level (Ancans et al., 2001; Zeng et al., 2017;). *OCA2* serves as a principal regulator of melanosome pH and plays a crucial role in modulating anion channels essential for pigmentation (Bellono et al., 2014). This gene is significantly associated with variations in human skin, hair, and eye color. Individuals with *OCA2* dysfunction exhibit markedly reduced pigmentation levels (Le et al., 2014). Moreover, *OCA2* plays an important role in the classification and transport of melanosomes (Sitaram and Marks, 2012). The findings of this study align with prior research, which demonstrated that the expression level of the *OCA2* gene and melanin deposition in dark black webbed feet were significantly higher compared to those in light black and colorless webbed feet. This observation suggests that the *OCA2* gene plays a pivotal role in determining the pigmentation of geese webbed feet. Whether the *OCA2* gene is central to the pigmentation of goose webbed feet and its underlying molecular mechanisms requires further verification and investigation.

Additionally, the metabolomic analysis revealed that L-tyrosine and 5,6-dihydroxy-2-indole carboxylic acid, key intermediates in the melanin biosynthesis pathway associated with tyrosine metabolism, were significantly enriched in pigmented webbed feet. As a precursor for melanin synthesis, L-tyrosine levels were notably higher in colorless webbed feet compared to dark black webbed feet. This discrepancy likely arises from the active melanin synthesis in dark black webbed feet, which consumes greater amounts of L-tyrosine. Conversely, the inhibition of melanin synthesis in colorless webbed feet leads to the accumulation of L-tyrosine. These findings suggest that the absence of pigmentation in colorless webbed feet may be due to an obstruction in melanin production rather than a deficiency in L-tyrosine supply. L-tyrosine serves as the primary precursor for melanin biosynthesis. During melanin formation, L-tyrosine undergoes enzymatic catalysis by *TYR, TYRP1*, and *DCT*, key enzymes in the melanin synthesis pathway (Galvan et al., 2016; Finlay et al., 2016). Transcriptome analysis revealed that the expression levels of *TYR, TYRP1*, and *DCT* genes were significantly elevated in pigmented webbed feet compared to non-pigmented webbed feet, indicating higher melanin synthesis activity in pigmented webbed feet. Consequently, this results in a lower abundance of L-tyrosine in pigmented webbed feet. Notably, compared to colorless webbed feet, the concentration of 5,6-dihydroxy-2-indole carboxylic acid, a downstream metabolite of tyrosine metabolism, was significantly elevated in dark webbed feet. This suggests that the metabolic conversion of tyrosine into melanin intermediates is more active in these webbed feet, thereby enhancing pigmentation. Further analysis revealed that key genes involved in melanin biosynthesis, including *TYRP1, TYR*, and *DCT*, were significantly positively correlated with melanin synthesis intermediates such as 5,6-dihydroxy-2-indole carboxylic acid.

In summary, this study reveals the significant correlation between goose webbed feet color and melanin biosynthesis. The expression of melanin in the webbed feet is regulated by several genes, including *TYRP1, PMEL, DCT, TYR, OCA2, MC1R, RAB38, WNT16, CAMK2A*, and *MLANA*, with particular emphasis on the pivotal role of the *OCA2* gene in modulating the intensity of melanin deposition. Metabolomics data further substantiate that the coloration of goose webbed feet is closely associated with two key metabolites: L-tyrosine and 5,6-dihydroxyindole carboxylic acid. This research offers novel insights into the molecular regulatory mechanisms underlying phenotypic diversity in avian epidermal tissues.

## Conclusion

This study systematically elucidated the molecular metabolic regulatory mechanism underlying goose webbed feet pigmentation. Histological analysis revealed that the melanin density in dark black webbed feet was significantly higher than in colorless webbed feet, where almost no melanin deposition was detected. Transcriptomic profiling identified 10 key genes that were specifically highly expressed in pigmented tissues and were significantly enriched in the melanin synthesis pathway. Metabolomic data analysis demonstrated that pigmented tissues accumulated intermediates of melanin synthesis, whereas colorless webbed feet exhibited an accumulation of L-tyrosine, suggesting a potential blockage in the melanin synthesis pathway. More importantly, this study highlights for the first time the pivotal role of the *OCA2* gene within the pigment regulatory network. This research provides multi-omics evidence supporting the mechanism of pigment formation and suggests that future investigations focus on the molecular regulatory pathways and functional mechanisms of the *OCA2* gene.

## Author contributions

Yi Liu and Kaiqi Weng contributed equally to this work. Yi Liu, Kaiqi Weng, Guangquan Li, Huiying Wang, Yu Tan and Daqian He conceived and designed the study. Yi Liu, Guangquan Li, Yu Tan and Huiying Wang performed the experiments and analyzed the data. Yi Liu, Kaiqi Weng, and Guangquan Li wrote the manuscript. Daqian He reviewed and edited the manuscript. All the authors have read and approved the final manuscript.

## Declaration of competing interest

The authors declare that they have no known competing financial interests or personal relationships that could have appeared to influence the work reported in this paper.

## Appendix Supplementary materials

Image, application 1

Image, application 2

## Footnotes

Supplementary material associated with this article can be found, in the online version, at doi:10.1016/j.psj.2025.105233.
