# Supplementary material for: Genetic and metabolic mechanisms underlying webbed feet pigmentation in geese: Insights from histological, transcriptomic, and metabolomic analyses
Source: Poult Sci. 2025 Apr 29;104(8):105233. doi: 10.1016/j.psj.2025.105233 (PMC12142325; doi:10.1016/j.psj.2025.105233)
Supplement: Supplementary file 1 [file mmc1.docx]

**Additional table 1.** Real-time PCR primer sequences.

| Gene Name | Primer SequenCes (5’–3’) | Annealing Temperature | Size of target fragments |
| --- | --- | --- | --- |
| *OCA2* | F:CCTTGTTGTTCTGGTCTCCTT  R:TTCACTCGGCTCCTTATTGTAA | 60 ℃ | 79 bp |
| *WNT16* | F:TACAGATATCAGACAGACT  R:TCTTCTACACAGTAATTGG | 60 ℃ | 121 bp |
| *TYRP1* | F:AATGAGATGTTTGTTACTG  R:ACTGATCAGTGAGAAGAGG | 60 ℃ | 208 bp |
| *TYR* | F:GCGACTGAGAACGAGAAGAA  R:AAGAGTGTGTCCCGAGAGGC | 60 ℃ | 222 bp |
| *DCT* | F:CCGCAATTCCAGTTTCAGCT  R:ACCGCTTCATCCACTCATCA | 60 ℃ | 209 bp |
| *CAMK2A* | F:GCACACGACGATCCTGAAC  R:TGATGCGGATGTAGGCGAT | 60 ℃ | 74 bp |
| *PMEL* | F:GTCGTCTACCACTACCGCG  R:ACCTGGTCTGTGATGCTGAA | 60 ℃ | 80 bp |
| *MC1R* | F:CTACCACAGCATCATGACGC  R:GCATGAAGAGGAAGAAGCCG | 60 ℃ | 146 bp |
| *GAPDH* | F:GGTGGTGCTAAGCGTGTCAT  R:CCCTCCACAATGCCAAAGTT | 60 ℃ | 200 bp |
