# Supplementary material for: Genetic and metabolic mechanisms underlying webbed feet pigmentation in geese: Insights from histological, transcriptomic, and metabolomic analyses
Source: Poult Sci. 2025 Apr 29;104(8):105233. doi: 10.1016/j.psj.2025.105233 (PMC12142325; doi:10.1016/j.psj.2025.105233)
Supplement: Supplementary file 2 [file mmc2.docx]

**Additional table 2.** *OCA2* gene in situ hybridization probe sequences.

| Probe name | Probe SequenCes (5’–3’) |
| --- | --- |
| *OCA2* | CCGGTTTGGATGCCACTGTTGCAG |
|  | GAAGCTTTCCAGAGGCGAGACAGC |
|  | TTCTGCAATCAGGACATGCCTGGG |
|  | GCGCTGTGCAGTCAACCTCCAAAC |
|  | GAAGGGTTGCCCATTCCACTCGGT |
